# Editorial: Minimally invasive cardiothoracic surgery: cost-effectiveness, prognostic factors, and outcomes

**DOI:** 10.3389/fsurg.2024.1482274

**Published:** 2024-09-03

**Authors:** Mohamed Rahouma, Massimo Baudo, Akshay Kumar, Magdy El-Sayed Ahmed

**Affiliations:** ^1^ Cardiothoracic Surgery Department, Weill Cornell Medical Center, New York-Presbyterian, New York, NY, United States; ^2^ Surgical Oncology Department, National Cancer Institute, Cairo University, Cairo, Egypt; ^3^ Department of Cardiac Surgery Research, Lankenau Institute for Medical Research, Wynnewood, PA, United States; ^4^ Department of Cardiothoracic Surgery, NYU Langone Health, New York, NY, United States; ^5^ Cardiothoracic Surgery Department, Mayo Clinic, Jacksonville, FL, United States; ^6^ Department of Surgery, Faculty of Medicine, Zagazig University, Zagazig, Egypt

**Keywords:** minimally invasive, cardiothoracic surgery, prognostic factors, esophageal cancer, NSCLC, lymph node harvest

**Editorial on the Research Topic**
Minimally invasive cardiothoracic surgery: cost-effectiveness, prognostic factors, and outcomes

Over the years, minimally invasive cardiothoracic surgery (MICTS) has gained significant traction, largely due to its benefits in reducing postoperative pain, lower risk of infection and hospital length of stay (1, 2). Despite initial concerns regarding the challenges of limited exposure in complex procedures, longer operative times, and patient safety, the refinement of surgical techniques and the development of specialized tools have made MICTS a widely accepted alternative to traditional open surgery (3). Current research highlights the long-term effectiveness and safety of MICTS, demonstrating that major cardiothoracic operations can be performed with outcomes comparable to open surgery (1, 2). The feasibility of MICTS varies among patients. Thus, underscoring the need for careful selection based on individual prognostic factors in the context of personalized medicine is important.

This research topic in Frontiers in Surgery comes to shed light on the surgical outcomes, prognostic factors, and cost-effectiveness of MICTS including mini-thoracotomy, mini-sternotomy, video assisted thoracoscopic surgery (VATS), and robotic-assisted surgery. This topic includes 6 manuscripts (1 mini-review and 5 original research articles).

In their study on non-small cell lung cancer (NSCLC), Hurley et al. compared robotic-assisted and VATS lymph node dissection and showed that in robotic operations lymph node dissection was more extensive compared to VATS (*p* = 0.0002). This was in line with the recent ROMAN, RAVAL, and RVLob randomized trials (4–6). However, evidence is still controversial in literature, as highlighted by a meta-analysis of retrospective studies that suggested no significant differences between the two approaches (7). This crucial topic in NSCLC among others was reviewed by Patel and Bille in their mini-review on lymph node dissection in lung cancer surgery (Patel and Brille). Their paper focuses on the debate over the best approach to lymph node assessment in lung cancer and that recent studies show no significant survival difference between mediastinal lymph node dissection and nodal sampling. As minimally invasive techniques like robotic surgery advance, they promise improved lymph node sampling and outcomes, but the ideal lymph node resection strategy remains a topic of ongoing debate.

Hu et al. reported in their work that five patients with traumatic flail chest were treated with a new 3D printed external fixation guide combined with VATS. All patients had successful operations, each lasting less than an hour, and experienced minimal blood loss. Within 6 h postoperatively, the patients were able to get out of bed and move around, reporting a significant reduction in chest pain and a substantial improvement in their ability to cough. Additionally, their results demonstrated a thoracic volume recovery rate of around 90%, resolving atelectasis and correcting restrictive ventilation dysfunction.

Two included articles analyzed predictors in esophageal cancer surgery (Tupper et al., Zhang et al.). Tupper et al. showed in their adjusted multivariable logistic regression that there was a 19% increase in 1-year mortality odds and 39% increase in anastomotic leak odds for every additional operative hour. Zhang et al. reported that hybrid/open esophagectomy, longer operation time, intraoperative blood transfusions, and prognostic nutritional index were independently associated with unplanned intensive care unit admission. Besides, at subgroup analysis minimally invasive surgery was associated with lower rates of intraoperative blood transfusions.

Finally, the paper by Li et al. analyzed hemodynamic changes of left subclavian artery (LSA) after simulating the covering half of the ostium by thoracic endovascular aortic repair through computational fluid dynamics. Their research showed that partially covering the LSA ostium reduces blood flow, velocity, and wall shear stress, potentially accelerating arteriosclerosis in the LSA due to hemodynamic changes. Furthermore, this partial coverage causes turbulent flow and increased vascular pressure at the orifice, which may damage the arterial endothelium and heighten the risk of arteriosclerosis. Additionally, the turbulence and low-velocity zones behind the stent membrane could lead to local acute thrombosis.

The articles included in this research topic provided interesting updates in different cardiothoracic surgeries regarding possible predicting factors that need further clinical evaluation to be validated. Nowadays, minimally invasive surgery is increasingly considered in cardiothoracic surgery, but certain drawbacks associated with this technique must be carefully weighed when selecting the appropriate surgical approach for each patient.
